# Editorial: Aging and the oocyte

**DOI:** 10.3389/fendo.2024.1361115

**Published:** 2024-01-16

**Authors:** Yun-Yao Luo, Guo-Ning Huang, Jing-Yu Li

**Affiliations:** ^1^Chongqing Key Laboratory of Human Embryo Engineering, Center for Reproductive Medicine, Women and Children’s Hospital of Chongqing Medical University, Chongqing, China; ^2^Chongqing Clinical Research Center for Reproductive Medicine, Chongqing Health Center for Women and Children, Chongqing, China

**Keywords:** aging, oocyte, maternal age, oxidative stress, follicular microenvironment, brown adipose tissue, fetal malformations

On a global scale, the rising average age of women at childbirth has emerged as a persistent trend. The fertility of women typically diminishes with advancing age, and it experiences a significant decline after reaching the age of 38. The main factor contributing to the decline in fertility is the decrease in oocyte quality associated with aging. Emerging evidence has shown that the deterioration of oocyte quality primarily arises from mitochondrial dysfunction, oxidative stress, and heightened chromosomal aneuploidy. These factors contribute to a reduction in oocyte developmental potential, as well as a decrease in fertilization rate and embryo developmental block.

To begin, we want to draw readers’ attention to a comprehensive review on the effect of oxidative stress (OS) on ovarian aging by Shi et al. They described OS level in premature ovarian insufficiency (POI) patients. OS has the potential to adversely affect multiple systems within the body, including the reproductive system. It impacts sperm, eggs, embryos, and the endometrium, thereby influencing embryo implantation, which could be a contributing factor to the decrease in female fertility. As individuals age, there is a decrease in the body’s capacity to counteract the effects of OS, leading to an accumulation of excessive levels of reactive oxygen species (ROS). The role of ROS is significant in follicle growth, intrinsic angiogenesis, and sex hormone synthesis. Shi et al., discussed the correlation between OS and POI and determined that OS has the potential to modulate genetic material, signaling pathways, transcription factors, and the ovarian microenvironment. This can result in aberrant apoptosis of ovarian granulosa cells, abnormal meiosis, and decreased mitochondrial DNA, consequently hastening the progression of ovarian aging. Antioxidants such as melatonin, coenzyme Q, and resveratrol, as well as mesenchymal stem cells and biological enzymes, have been found to delay the progression of POI by reducing the levels of ROS in the body. Furthermore, we would want to draw attention to the way that aging-related modifications to the follicular microenvironment affect the deterioration of oocyte quality. Nitric oxide (NO) has been demonstrated to be a pervasive molecule within the oocyte microenvironment, playing a role in preserving oocyte quality and ensuring the optimal fertilization window for oocytes. Goud et al., conducted a study that examined the hypothesis suggesting a correlation between chronological aging and a deficiency in NO as well as an increase in ROS within the oocyte microenvironment. Their study indicated that supplementation with an NO-donor may alleviate oocyte aging by improving zona pellucida hardening, altering spindle and ooplasmic microtubules, and the meiosis process.

Researchers have been dedicated to exploring methods for enhancing the quality of aging oocytes. In recent years, brown adipose tissue (BAT) transplantation has demonstrated success as a method for treating age-related diseases. The most recent study has determined that BAT transplantation has the potential to enhance the quality of follicles and oocytes in aged mice (1, 2). In a study by Zhang et al., the impact of BAT on the quality of aging oocytes was assessed *in vivo*. The author utilized BAT-derived exosomes as the primary vehicle for long-distance signal transduction to other cells. Upon administration of BAT-derived exosomes to aged mice, it was observed that these exosomes have the capacity to augment the mitochondrial function of aging oocytes, support follicle survival, extend ovarian lifespan, and enhance the fertility of aged mice.

Moving from animal to human data, advanced maternal age is associated with diminished fertility, leading many aged women to turn to assisted reproductive technology (ART). However, this demographic faces an elevated risk of early pregnancy loss and reduced live birth rates when undergoing assisted reproduction. Yang et al., conducted a retrospective analysis of a substantial database from a single institution to investigate the impact of different proportions of follicle size on oocyte and embryo quality in aged patients. The author proposed that age has an adverse impact on the outcomes of ART. To achieve better ART outcomes, in aged patients (≥ 35 years), it is preferable to trigger when the proportion of medium follicles (diameter 16-18 mm) is equal to or greater than that of small follicles (< 16 mm). Their findings offer valuable insights for tailoring personalized treatment plans in aged women.

In the end, our attention will be directed towards instances of maternal age and fetal malformation. Zhang et al., conducted a cross-sectional survey with the aim of identifying potential gender bias in cases of fetal malformations. The authors performed genetic testing and documented a positive correlation between the incidence of genetic factors and maternal age.

Maternal age is the primary factor contributing to diminished oocyte quality. In addressing this issue, the limited effectiveness and efficacy of existing clinical treatment methods are attributed to the unchangeable nature of age and the unclear molecular mechanism involved. We aim to stimulate readers’ critical thinking and inspire new ideas for future research directions through this Research Topic, which offers deeper insight into oocyte aging.

## Author contributions

Y-YL: Funding acquisition, Writing – original draft, Writing – review & editing. G-NH: Supervision, Writing – original draft, Writing – review & editing. J-YL: Supervision, Writing – original draft, Writing – review & editing.
